# Disease severity classification using passively collected smartphone-based keystroke dynamics within multiple sclerosis

**DOI:** 10.1038/s41598-023-28990-6

**Published:** 2023-02-01

**Authors:** Aleide Hoeijmakers, Giovanni Licitra, Kim Meijer, Ka-Hoo Lam, Pam Molenaar, Eva Strijbis, Joep Killestein

**Affiliations:** 1 Neurocast B.V., Amsterdam, The Netherlands; 2 Department of Neurology, Amsterdam University Medical Centers, Amsterdam, The Netherlands

**Keywords:** Biomarkers, Multiple sclerosis

## Abstract

Multiple Sclerosis (MS) is a progressive demyelinating disease of the central nervous system characterised by a wide range of motor and non-motor symptoms. The level of disability of people with MS (pwMS) is based on a wide range of clinical measures, though their frequency of evaluation and inaccuracies coming from objective and self-reported evaluations limits these assessments. Alternatively, remote health monitoring through devices can offer a cost-efficient solution to gather more reliable, objective measures continuously. Measuring smartphone keyboard interactions is a promising tool since typing and, thus, keystroke dynamics are likely influenced by symptoms that pwMS can experience. Therefore, this paper aims to investigate whether keyboard interactions gathered on a person’s smartphone can provide insight into the clinical status of pwMS leveraging machine learning techniques. In total, 24 Healthy Controls (HC) and 102 pwMS were followed for one year. Next to continuous data generated via smartphone interactions, clinical outcome measures were collected and used as targets to train four independent multivariate binary classification pipelines in discerning pwMS versus HC and estimating the level of disease severity, manual dexterity and cognitive capabilities. The final models yielded an AUC-ROC in the hold-out set above 0.7, with the highest performance obtained in estimating the level of fine motor skills (AUC-ROC=0.753). These findings show that keyboard interactions combined with machine learning techniques can be used as an unobtrusive monitoring tool to estimate various levels of clinical disability in pwMS from daily activities and with a high frequency of sampling without increasing patient burden.

## Introduction

Multiple Sclerosis (MS) is a progressive and chronic disease of the central nervous system (CNS) and is the most common cause of non-traumatic disability in young adults^1^. Although the clinical course is highly variable, approximately 85% of people with MS are diagnosed with relapsing-remitting MS (RRMS), which is characterized by unpredictable relapse resulting in neurological deficits separated by periods of remission (i.e. apparent quiescence or stability of disease)^2^. People with RRMS may develop a progressive course of the disease secondary progressive MS (SPMS) with a gradual increase in disability with or without relapses within a few decades. primary progressive MS (PPMS) has an onset with gradual accumulation of disability^3^. The clinical spectrum of MS covers a wide range of motor and non-motor symptoms^4^. Motor symptoms are seen as the clinical hallmark of the disease and can present with changes in mobility and coordination of lower and upper limb extremities. On top of motor deficits, problems with cognition are frequently seen, and more generally, symptoms vary according to the location of active lesions in the CNS^4^.

The clinical status of people with MS is based on a wide range of clinical measures, including Magnetic Resonance Imaging (MRI) scans, the Expanded Disability Status Scale (EDSS), Multiple Sclerosis Functional Composite Score (MSFC) and self-reported questionnaires during a hospital visit^4,5^. However, these measures are limited by the evaluation frequency and are influenced by the expert’s subjective experience and self-reported measures. Conversely, objective measures coming from physical biometrics and captured via electronic devices can provide insight into the disease status and offers a cost-efficient addition to on-site clinical monitoring with the opportunity to monitor symptoms passively and support disease management^6^.

Nowadays, typing on keyboards is a common task carried out multiple times daily, requiring motors and non-motor functions, like eye/hand coordination, manual dexterity and cognition. Therefore, it is hypothesised that these “typing signatures” are influenced by alterations in motor and non-motor symptoms, as is frequently seen in neurological diseases such as MS and thus could provide insight into the status of a person at any given time. Recent studies have shown that such timing information associated with keystrokes, namely Keystroke Dynamics (KD), can be potentially used to detect fine motor skills decline in early-stage Parkinson’s disease^7^, psycho-motor impairment, such as sleep inertia^8^, and identification of depressive tendency^9^. Furthermore, KD have been found to be effective as a computer system protection while maintaining a high level of usability^10^.

Within an MS population, it has been shown that KD were significantly different between healthy individuals and people with MS, and were associated with clinical outcome measures, which quantifies manual dexterity, information processing speed, and clinical disability^11^. Additionally, KD have demonstrated a higher sensitivity to changes in disease activity, fatigue, and clinical disability compared to commonly used clinical measures via detection of important changes beyond measurement error on a group level^12^. Finally, the association between KD and clinical outcomes in longitudinal settings has been shown, namely that worse arm function corresponds with longer latencies in typing across and within patients, and worse processing speed corresponds with higher latencies relating to punctuations and backspaces across subjects^13^.

The current study investigates whether keystroke-related data combined with machine learning-based methods yield sufficient predictive power to discriminate between people with Multiple Sclerosis (pwMS) and a Healthy Control (HC) group and between different levels of disease severity, including clinical disability, manual dexterity and cognition. The data comes from an observational cohort study of 126 subjects (24HC/102MS patients) carried out at the Amsterdam University Medical Center, located at the VU University Medical Centre. This study included five clinical visits with three-month intervals for a total duration of 12 months. The keystroke-related data was collected passively by the *Neurokeys* App designed by the Dutch company Neurocast B.V.^14^. The Neurokeys App is a customized keyboard developed for Android and iOS that replaces the user’s native keyboard and allows to cache keyboard interactions of interest, namely alphanumeric keys, backspaces, space bars and punctuation keys. The time-stamped raw keystroke sequences were used to construct various statistical features of keystroke dynamics variables aggregated on a daily level. Keystroke features were further clustered into composite scores to reduce the number of input variables and consequently minimize overfitting issues.

## Material and methods

### Study procedures

The study protocol was approved by the Medisch-Ethische Toetsingscommissie Vrije Universiteit Medisch Centrum (medical-ethical committee, approval IRB reference 2017.576), and the institutional data protection officer conforming to the General Data Protection Regulation (GDPR). In compliance with Dutch legislation regarding clinical research involving medical devices, *Dutch Health and Youth Care Inspectorate* were notified of the study (reference VGR2006948). Subjects held the right to withdraw from the procedure without providing any justification. Written informed consent was obtained from all participants. Finally, the study was registered at trialregister.nl(NL7070) .

### Clinical outcomes

In this cohort study, several clinical outcomes widely used within an MS population were included: assessment of clinically reported relapses and conventional MRI for disease activity; EDSS, MSFC, patient-reported outcomes, quantitative MRI to evaluate domain-specific, overall severity of the disease and disease progression over time. As keystroke dynamics is most directly related to upper limb function and cognition, this work focus on the 3-monthly clinically assessed Nine Hole Peg Test (NHPT) and Symbol Digit Modalities Test (SDMT). Furthermore, the disease severity based on EDSS and clinical diagnosis (HC versus pwMS) are also analysed to quantify the prediction capabilities of keystroke dynamics on clinical disability.

The NHPT^15^ is a measure of manual dexterity, and the test consists in placing and removing nine pegs into and from nine holes using one hand. The test is performed twice for each hand, and the four trials are averaged into a single score (measured in seconds) where a higher score reflects poor performance, such as higher fine motor function impairment. The SDMT^16^ is a symbol substitution test that measures information processing speed, the cognitive domain that is most commonly affected and indicative of overall cognitive functioning in MS. The test consists of matching nine symbol-digit pairs within a 90-second trial. The final score is adimensional and lies between 0 and 105. The higher the score, the better the patient’s cognitive performance. Finally, the EDSS is used to quantify the overall disease severity in pwMS and monitors changes in the level of disability over time^17^. The EDSS assigns a functional system score in eight functional systems.As reported in the literature, the lower scale values (0-4.0) are influenced by impairments detected by the neurological exam of eight functional systems, while the values above 4.0 are mainly based on the walking ability, and values above 6 are mainly on patients’ handicaps^18^.

### Study design

An observational cohort study was conducted at Amsterdam University Medical Centres, location VU University Medical Centre. The study cohort comprised two groups of Dutch-speaking people, the MS patient group, which consisted of 102 subjects with MS and the HC group, which included 24 healthy subjects. Other inclusion criteria were regular smartphone usage on both iOS and Android and ages between 18 and 65. The exclusion criteria were an EDSS score of 7.5 or higher, clinical disease activity or changes in disease-modifying drugs in the past two months, significant visual or upper extremity deficits affecting the ability to type on a smartphone, and clinically significant mood, sleep, or behavioural disorders assessed via a screening physician^11^.

**Figure 1:**
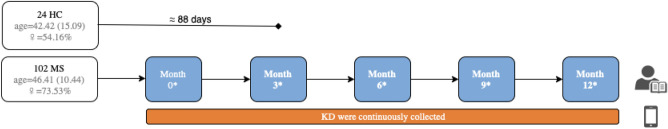
Graphical visualization of the study design. The MS patient group consisted of 102 subjects with MS and the HC group included 24 healthy subjects without any sign of MS. The study consisted of five clinical visits, collected at baseline (*m*00) and then at three-month intervals following baseline (*m*03, *m*06, *m*09, *m*12) for pwMS. Each clinical visit included the collection of the following clinical outcomes measures for the MS patient group: SDMT, NHPT, and EDSS.

The study consisted of five clinical visits collected at baseline (*m*00) and then at three-month intervals following baseline (*m*03, *m*06, *m*09, *m*12) for pwMS, only. For each clinical visit, the SDMT, NHPT and EDSS were collected, while HC collected on average 88 days of keystroke data starting from baseline (see Fig. 1). The level of cognitive disability was defined based on the SDMT cutoff proposed by Parmenter et al.^19^, where a value of $\textrm{SDMT}>$ 55 denotes a low level of cognitive deficit. Regarding both NHPT and EDSS, the scores were binarized using a median split approach. In this way, sufficient fine motor skills and a low disease severity are given by a $\textrm{NHPT} \le 20.40 s$ and $\textrm{EDSS} \le 3.5$, respectively. Any other score outside the above ranges is associated with low fine motor skills and high disease severity. Throughout the study, keyboard interaction data were remotely collected in a real-world environment using a mobile application, namely *Neurokeys*. The *Neurokeys* app^14^, available for Android and iOS, was installed on the participants’ phone to collect keystroke data in a real-world setting unobtrusively. *Neurokeys* consists of a software QWERTY keyboard designed similarly to the default keyboard with comparable functionalities, such as auto-correction and word suggestions. After the app installation, the default keyboard is replaced by the *Neurokeys* keyboard and data from each typing session are automatically gathered. The raw data contain timing information of pressing and releasing events during a typing session. Note that neither letters nor the corresponding (*x*, *y*) coordinates relative to the key presses are collected to guarantee the participant’s privacy. All data gathered were temporarily stored locally on the mobile device before being sent in batches to secure cloud storage whenever an internet connection was available.

### Feature engineering

Let us define $t_{n}^{p}$ and $t_{n}^{r}$ as the timestamp in milliseconds relative to the key press and release event, respectively. For each keystroke event, one can compute sequences of keystrokes that are purely related to the typing rhythm, precisely the time between a key is released and the next key press, the time a key is pressed, the time between successive key presses and the time between successive key releases, a.k.a. Flight Time ($\textrm{FT}_{n}$), Hold Time ($\textrm{HT}_{n}$), Press-Press Latency ($\textrm{PPL}_{n}$) and Release-Release Latency ($\textrm{RRL}_{n}$), respectively. These sequences can be expressed mathematically as follows:

1 $$\begin{aligned} \begin{aligned} \textrm{HT}_{n}&= t_{n}^{r} - t_{n}^{p}, \;\;\,\,n = 1,2, ..., N, \\ \textrm{FT}_{n}&= t_{n+1}^{p} - t_{n}^{r}, n = 1,2, ..., N-1, \\ \textrm{PPL}_{n}&= t_{n+1}^{p} - t_{n}^{p}, n = 1,2, ..., N-1, \\ \textrm{RRL}_{n}&= t_{n+1}^{r} - t_{n}^{r}, n = 1,2, ..., N-1. \end{aligned} \end{aligned}$$

where *N* is the amount of keys pressed during a specific interval, for example, daily, hourly, or session typing intervals. Similarly, one can construct additional keystroke sequences which are conditional to certain events, i.e. the flight time after a punctuation event a.k.a. After Punctuation Pause ($\textrm{APP}_{n}$) and the flight time prior to and post a backspace event, denoted as Pre-Correction Slowing ($\textrm{PreCS}_{n}$) and Post-Correction Slowing ($\textrm{PostCS}_{n}$), respectively (see supplementary material Table A2 for a summary table). Note that, prior to any further mathematical operation, $\textrm{FT}_{n}$ and $\textrm{HT}_{n}$ are filtered to avoid outliers from edge cases, such as when the keyboard is on-screen without any typing activity or when special characters are required. The continuously collected keystroke sequences are subsequently aggregated per day using several summary statistics shown in the supplementary material Table A1.

**Figure 2:**
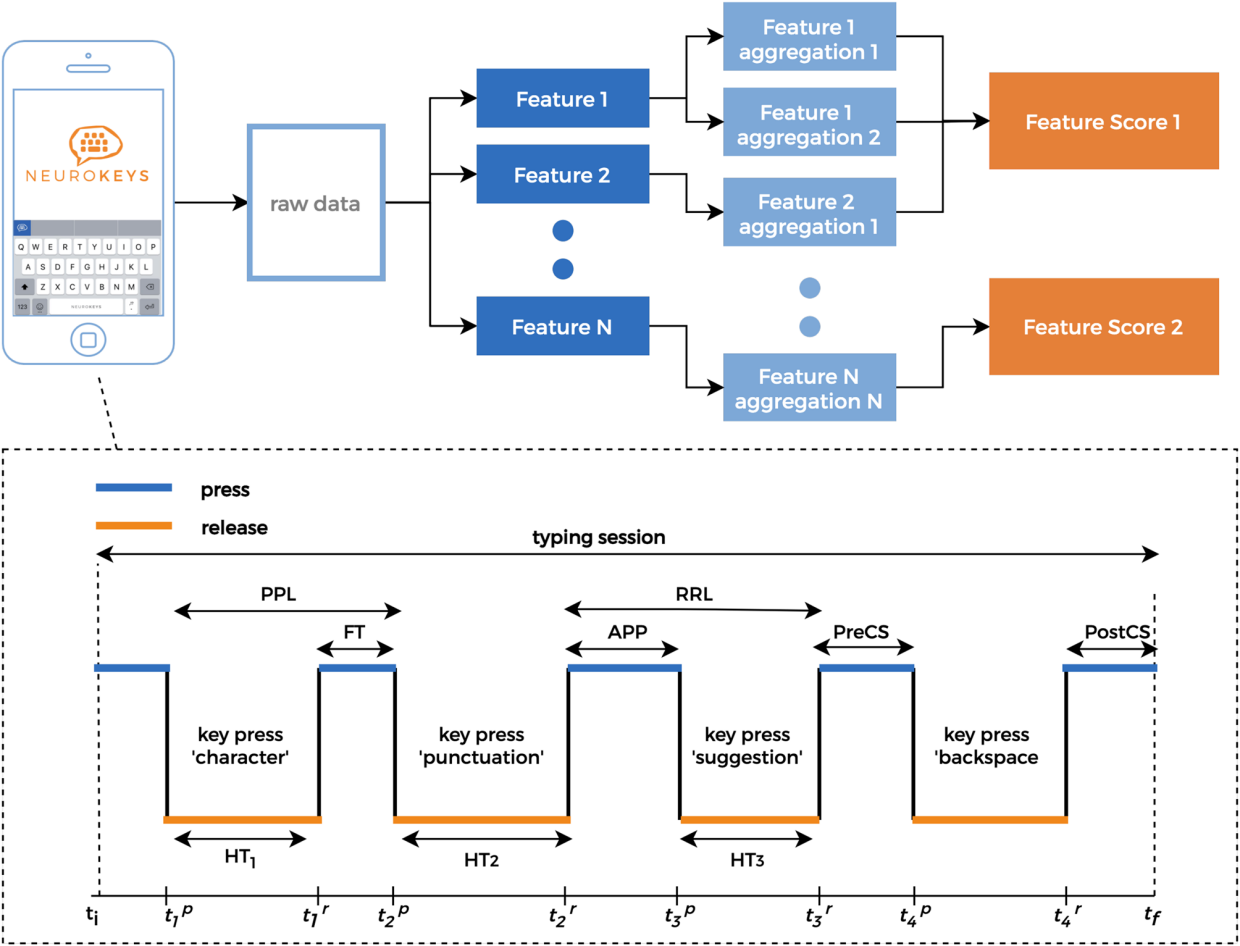
The keystroke data are continuously collected through the *Neurokeys* app. Various features are constructed from the raw data, and a graphical representation of keystroke sequences is provided. Here, the hold time keystroke variable $\textrm{HT}_{{1,2,3}}$ represents the time for which a key is pressed, whereas the flight time ($\textrm{FT}$) is the time between two key presses. The Pre-Correction Slowing ($\textrm{PreCS}$), Post-Correction Slowing ($\textrm{PostCS}_{1}$), and After Punctuation Pause ($\textrm{APP}_{1}$) are specific cases of flight time relative to the time before and after a backspace and the time after punctuation. After several preprocessing steps, the keystroke sequences are aggregated on a daily level using summary statistics such as mean and standard deviation (see supplementary material Table A1 for a complete list). Finally, composite scores are created by averaging subsets of keystroke features tuned based on both a hypothesis and data-driven approach^13^.

Finally, composite scores are created by averaging a cluster of features into single scores to reduce potential information overload^13^. More precisely, two Fine Motor Composite Score (FMCS), and a Cognition Composite Score (CCS) are derived, based on the hypothesis that timing-related features ($\textrm{PPL}_{n}$, $\textrm{RRL}_{n}$, $\textrm{HT}_{n}$, and $\textrm{FT}_{n}$) are more related to fine motor skills, while error-related ($\textrm{PreCS}_{n}$, and $\textrm{PostCS}_{n}$) and paralinguistic ($\textrm{APP}_{n}$) features are more related to cognitive processes. In addition to this theory-driven clustering, only highly correlated features were selected. Finally, besides keystroke sequences, additional features coming from counting the total number of events or relative to a specific event (e.g. the amount of times a user makes use of suggestion buttons) were constructed. Figure 2 graphically summarizes the keystroke data preprocessing pipeline introduced above.

### Feature selection

Several feature selection techniques were used to prune away non-useful features and to reduce the model’s complexity. First, features with a high percentage of missing values were discarded to avoid possible biases introduced by imputation methods. Missing values in keystroke data can arise whenever a user does not type within a specific time interval of interest, though they can also occur based on the individual typing style, e.g., a person that does not consistently use punctuations would lead to missing values in $\textrm{APP}$. Features with low variance are also discarded as they indicate low information content adding unnecessary computation burden^20^.

**Figure 3:**
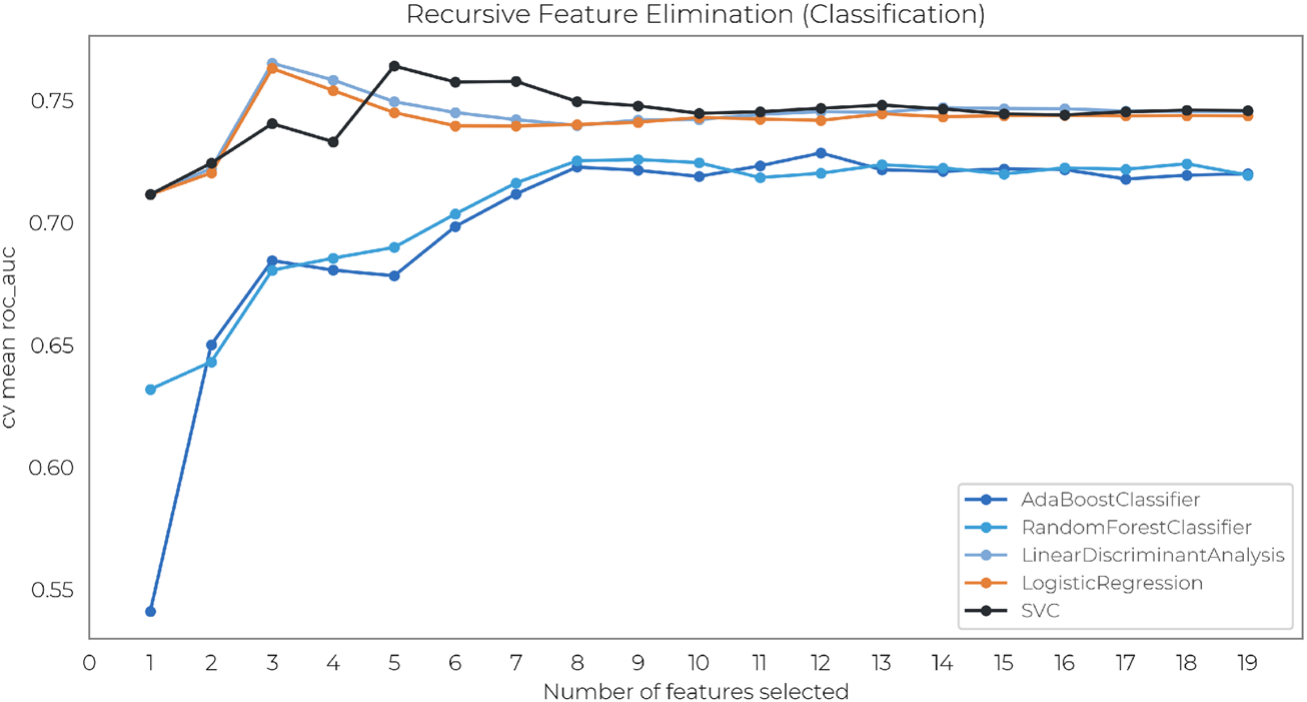
Example of feature selection using RFE schema with NHPT as a target. The *x*-axis shows the number of selected features, while the *y*-axis denotes the mean of AUC scores over k=5 folds for a given classifier. The final feature set is constructed by considering the most recurring features among all trained models where the mean AUC-ROC is maximized. One can also observe a performance plateau whenever more than eight features are used as predictors, regardless of the type of classifier.

The remaining features are evaluated using *wrapper methods*, namely via Recursive Feature Elimination (RFE). In short, RFE is a greedy optimization algorithm that aims to find the best performing feature set by repeatedly training models and keeping aside the best performing features at each iteration. Such a method relies on the machine learning model used; hence the best feature set will ultimately depend on the model architecture and the underlying cost function used during the training phase. In this work, five different estimators were separately trained using the RFE schema that selects the optimal features based on the Area Under the Receiver Operating Characteristic (AUC-ROC) and using a *group K-fold* cross-validation scheme with $k=5$. The final feature set for a given target was obtained by considering only the features yielding the best average AUC-ROC in cross-validation across all classifiers. An example of this procedure is graphically shown in Fig. 3 using the NHPT class as target.

### Classification pipeline and performance evaluation

Before any analysis and model training, the dataset is randomized and divided into a hold-out (20%) and training set (80%) with non-overlapping groups so that the same user will not appear in two different sets, preventing overestimating the generalization error due to data leakage issues^21^. The model selection and hyper-parameter tuning were carried out in the training set using a Leave-One-Group-Out Cross-Validation (LOGOCV) scheme where all samples from the $i_th$ subject are left out and used for each iteration for performance evaluation. In contrast, the remaining $N-1$ subjects are used to train and optimize a three-stage multivariate classification model.

**Figure 4:**
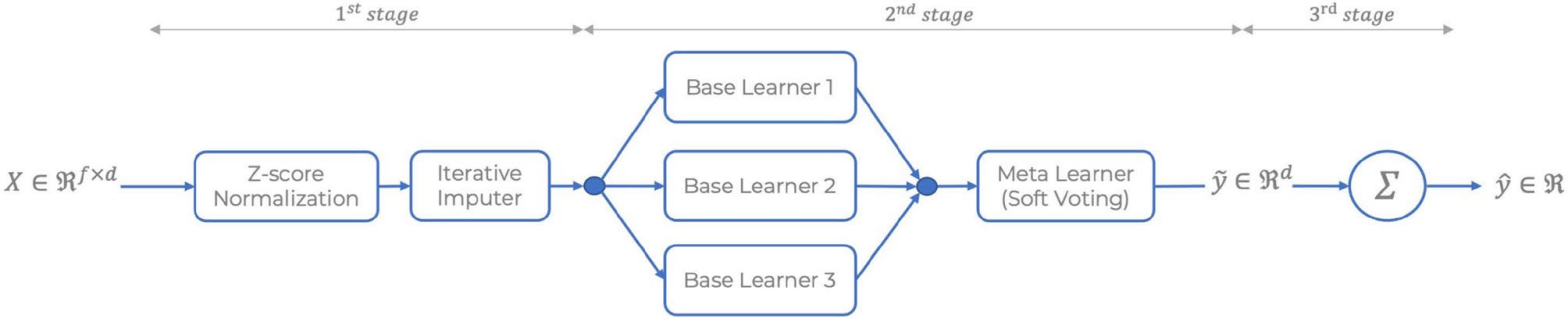
The classification pipeline for a given clinical outcome inference is a three-stage non-linear mapping, formally $\Phi : \Re ^{f \times d} \rightarrow \Re$. The pipeline requires a matrix containing *f* composite scores generated over *d* consecutive days as an input for a single subject. During the learning phase, the time window of length *d* is centred around the clinical visit where EDSS, NHPT, and SDMT scores are recorded. The matrix values are subsequently normalized, and missing values (due to insufficient data within a day) are imputed via chained equations^22,23^, a.k.a. Iterative Imputer. The second stage pipeline delivers an output $\tilde{y} \in \Re ^{d}$ with *d* predicted probabilities coming from an ensemble model composed of three classifiers (two for the clinical diagnosis) pointing to a soft voting meta learner. The third and last stage of the pipeline yields the actual prediction denoted as $\hat{y} \in \Re$ obtained by averaging the probabilities, i.e. $\hat{y} = \sum _{i = 1}^{d} \tilde{y}_{i}$. Depending the target and the corresponding feature set, $\hat{y}$ provides the estimation relative to the clinical diagnosis (HC versus pwMS), disease severity level based on EDSS, manual dexterity and cognitive function level based on NHPT and SDMT, respectively.

Regarding the model architecture, the first stage consists of an iterative imputer^22,23^ preceded by a z-score normalization. The second stage is an ensemble algorithm that combines the prediction probabilities of multiple and independent classifiers into one outcome, which aims to reduce further the generalization error^24^. In this stage, a prediction probability is derived for each daily aggregated keystroke feature, which yields sufficient typing events and lies within a predefined time window centred around the clinical visit. Note that the threshold relative to the minimum amount of daily typing events $\tau _{d}$ and the time windows $w_{d}$ were considered unknown variables and optimized using a typical hyper-parameter tuning procedure; hence both values were tailored for each target. The prognosis of the subject’s status is computed in the third and last stage by averaging the probabilities from the previous step. An illustration of the classification pipeline is depicted in Fig. 4. The classification pipelines are optimized with respect to the AUC-ROC; however, additional metrics are also supplied. A logistic regression test is conducted by regressing the binarized subject’s status on the prediction probability (outputted by the best-performing pipeline), demographic variables, and the daily average keystroke collected to assess their association and corresponding strength. Finally, the output of each machine learning model and features set is explained using SHapley Additive exPlanations (SHAP)^25^.

## Results

**Figure 5:**
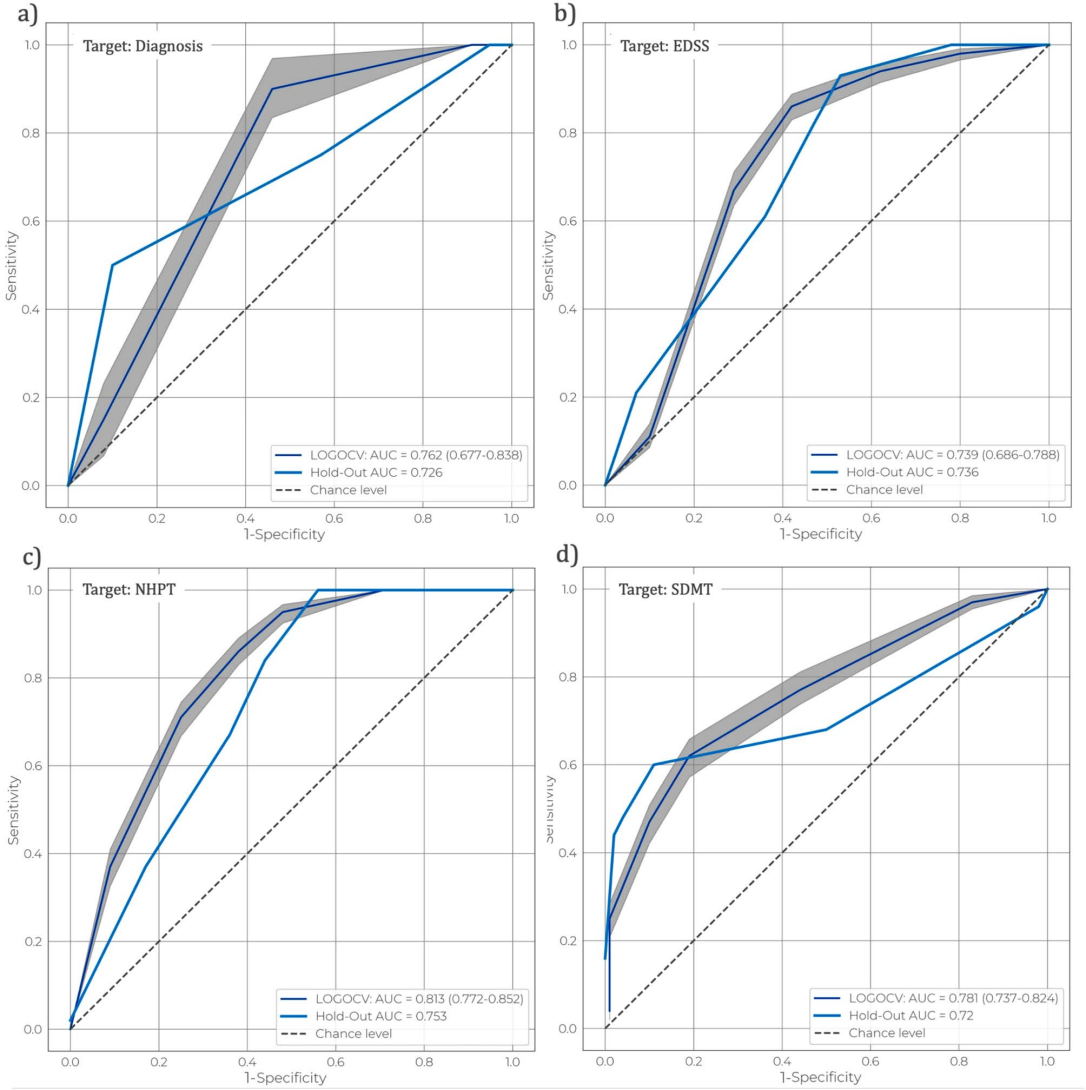
The mean AUC-ROC with the 95% confidence interval, computed over 1.000 bootstraps within the LOGOCV set, and the corresponding AUC-ROC carried out in the hold-out set, both coming from the best performing pipeline relative to each clinical outcome.

In total, 102 pwMS and 24 HC were included of whom demographical and clinical characteristics are summarised in Table 1 and supplementary material Table A4. The retention rate of patients with active keyboard use was 83.3% (for further information, refer to^13^). For the discrimination between pwMS and HC, an ensemble of two models was the best performing, consisting of a Random Forest (RF) and a Logistic Regression (LR), both trained with a balanced sample weight in order to counteract the class imbalance^26^. The cross-validation (CV) set yielded an AUC=0.762 [0.677-0.838; 95% CI] and a AUC-ROC=0.726 with 0.750/0.429/0.48 sensitivity/specificity/accuracy with a prevalence=0.16 in the Hold Out (HO) set. Regarding the estimation of the overall disability level quantified by the EDSS score, the best performing ensemble model consisted of a RF, a LR and a Quadratic Discriminant Analysis (QDA). An AUC=0.739 [0.686-0.788; 95% CI] was measured within the LOGOCV, while a AUC-ROC=0.736 with 0.821/0.533/0.644 sensitivity/specificity/accuracy with a prevalence=0.384 was obtained in HO set. The best classification pipeline for predicting fine motor skills based on NHPT consisted of a LR, a Gaussian Naive Bayes (GNB) and a Support Vector Machine (SVM). For this target, an AUC-ROC=0.813 [0.772-0.852; 95% CI] in LOGOCV and in HO set a AUC-ROC=0.753 with 0.837/0.556/0.709 sensitivity/specificity/accuracy with a prevalence=0.544 was obtained. Finally, the best performing machine learning pipeline for classifying the level of cognition deficit based on SDMT was a voting ensemble consisting of three models, a LR, a K-Nearest Neighbors (KNN) and a SVM. The model achieved an AUC-ROC=0.781 [0.737-0.824; 95% CI] in CV and AUC-ROC=0.720 with 0.600/0.891/0.789 sensitivity/specificity/accuracy with a prevalence=0.352 in the HO set. The Receiver Operating Characteristic (ROC) curves showing the performance of each classification pipeline for both the LOGOCV and HO set are presented in Fig. 5. The results provided in Table 2 show that the prediction probabilities generated by the classification pipelines are significantly associated with the corresponding clinical outcomes. Furthermore, a non-negligible relationship was also found, namely the level of education with both the EDSS and NHPT and age with the EDSS.

**Table 1:** Summary of the study cohort, demographic and clinical characteristics concerning each group (HC and pwMS). Clinical outcomes are also provided and shown solely for pwMS since such assessments were not carried out on healthy subjects. More information regarding the education demographics can be found in the supplementary material Table A4.

	HC (*n* = 24)	pwMS (*n* = 102)	*p* value
Demographics			
Age, years, mean (SD)	42.42 (15.09)	46.41 (10.44)	$0.248^a$
Sex, *n* (%)			$0.085^b$
Female	13 (54.16)	75 (73.53)	
Male	11 (45.84)	27 (26.47)	
Level of education, *n* (%)			$0.233^b$
Low	5 (20.83)	37 (36.27)	
Middle	11 (45.83)	46 (45.09)	
High	8 (33.33)	19 (18.62)	
MS type, *n* (%)			n.a.
Relapsing-remitting	n.a.	61 (59.80)	
Secondary progressive	n.a.	30 (29.41)	
Primary progressive	n.a.	11 (10.79)	
Clinical outcomes			
EDSS, median (IQR) [*n*]	n.a.	3.50 (2.5 – 4.5)[387]	n.a.
NHPT, median (IQR) [*n*]	n.a.	20.40 (18.6 – 22.6) [418]	n.a.
SDMT, median (IQR) [*n*]	n.a.	59.00 (50.0 – 66.0) [419]	n.a.

*HC* healthy controls;* pwMS* people with multiple sclerosis;* EDSS* expanded disability status scale;* NHPT* nine-hole peg test;* SDMT* symbol digit modalities test;* SD* standard deviation;* IQR* interquartile range.

^a^ Independent t-test.

^b^ Fisher’s Exact test.

**Table 2:** Results of the Logistic Regression test. The demographic variables (age, gender and education), the average of the daily keystroke and the prediction probability of the best performing model are treated as independent variables, while binarized clinical outcomes are considered dependent variables. Results show that the prediction probabilities of all models exhibit a statistically significant association with the subjects’ status.

	**Diagnosis**		**EDSS**		**NHPT**		**SDMT**	
	*coef*	*significance (p-value)*	*coef*	*significance (p-value)*	*coef*	*significance (p-value)*	*coef*	*significance (p-value)*
Age	$-$0.0244	(*p*=0.925)	**1.2126**	sig. (*p*<0.05)	0.0940	n.s. (*p*=0.793)	0.1101	n.s. (*p*=0.774)
Education	0.2970	(*p*=0.178)	**−0.6623**	sig. (*p*<0.05)	**0.7062**	sig. (*p*<0.05)	$-$0.2395	n.s. (*p*=0.358)
Gender	$-$0.3259	(*p*=0.152)	$-$0.0931	n.s. (*p*=0.769)	0.6346	n.s. (*p*=0.051)	$-$0.2243	n.s (*p*=0.457)
Daily Keystroke	$-$0.0321	(*p*=0.891)	0.5128	n.s. (*p*=0.090)	$-$0.4434	n.s. (*p*=0.164)	0.2380	n.s (*p*=0.390)
Prediction	**0.5753**	(*p*<0.05)	**0.7342**	sig. (*p*<0.05)	**1.4200**	sig. (*p*<0.001)	**1.1028**	sig. (*p*<0.05)

Significant value are in value [bold].

## Discussion

The findings of this study show that remotely collected keystroke interactions can potentially be used to discriminate between pwMS and HC and between different levels of clinical disability assessed by commonly used clinical outcome measures, including the disability status, upper limb function and information processing speed. Although typing on a smartphone is a very common and daily activity, it requires a broad range of upper extremity motor and visual skills to perform coordinated and successive hand/finger movements. Problems with these required skills are common in people with MS, including upper extremity motor coordination^27–29^, eye-hand coordination^28^, and manual dexterity^28,30^. Next to the motor skills, all cognitive skills are involved in typing behaviour, including attention and information processing speed. Problems with these two cognitive domains are commonly seen and present early in the disease^31–33^.

**Figure 6:**
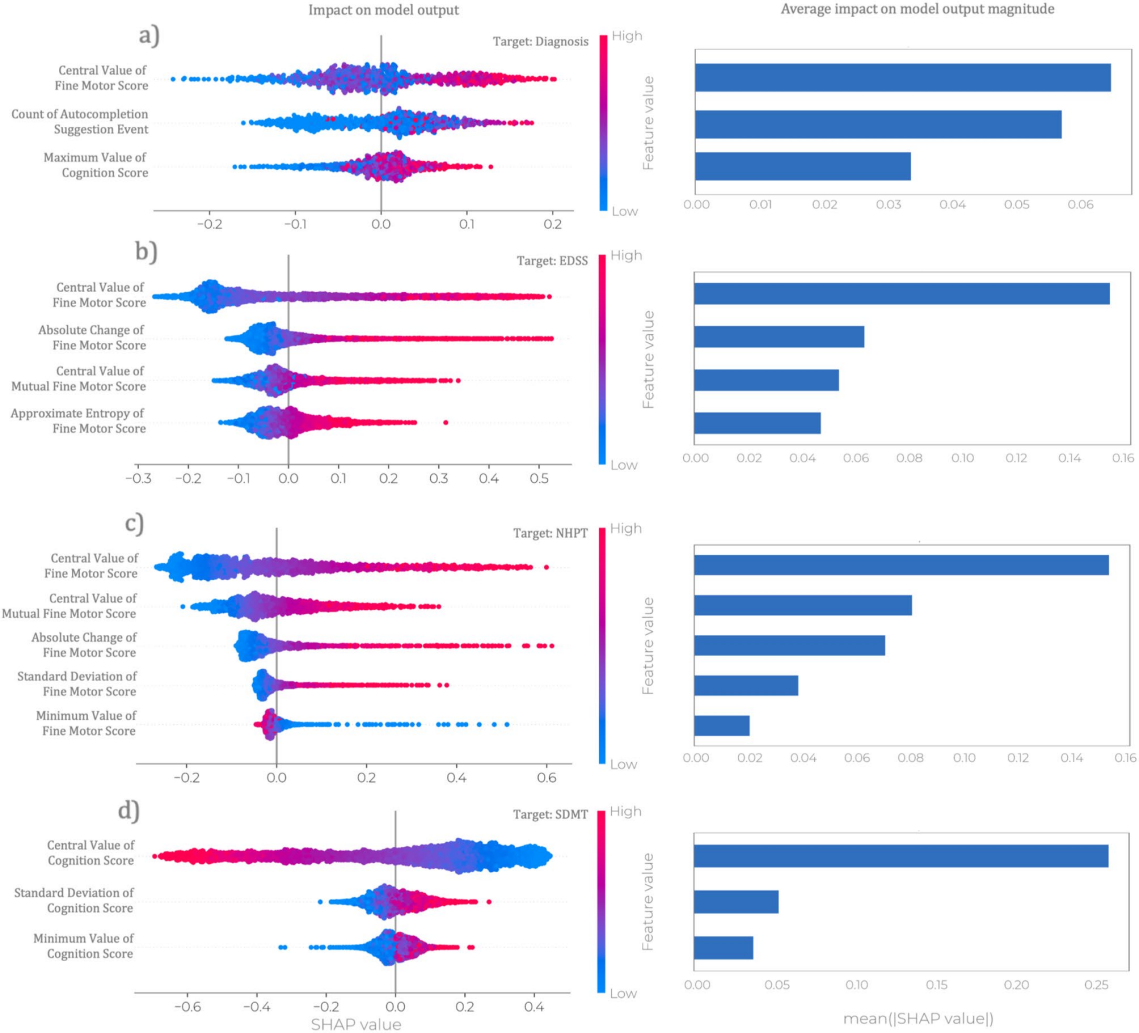
Model Interpretation using SHapley Additive exPlanations (SHAP). The left column provides the summary plots relative to the impact of features on the model prediction. Each point on the summary plot denotes a Shapley value for a given feature and an instance, while the colour represents the value of the feature from low to high. Overlapping points are jittered in y-axis direction to provide insight into the distribution of the Shapley values per feature. The right column provides the global importance of each predictor, which is achieved by averaging the absolute Shapley values per feature across the daily samples.

The study’s findings show that pwMS had different typing profiles than HC, probably driven by the symptoms pwMS experience. The extent to which specific functions are affected by the disease is assessed during a clinical visit by using a wide variety of clinical outcome measures. Four independent machine learning-based algorithms that leverage passively collected smartphone keystroke dynamics have been trained in discerning pwMS versus HC, estimating the level of disease severity, manual dexterity and cognitive capabilities. The interpretation of the prediction models’ output is addressed using the SHAP framework^25^ and shown in Fig. 6. In short this method indicates how much each predictor contributes, either positively or negatively, to the target variable with respect to the expected value of the target. supplementary material Table A3 lists the predictors used in this work with their respective keystroke features and aggregation type.

The discrimination between pwMS and HC is derived using three predictors: a time-related cluster, a cognitive-related cluster and the number of times a subject uses one of the three suggested words provided by a word suggestions implemented within the *Neurokeys* app. According to the proposed model, pwMS tend to type slower, have longer maximum latencies before and after correcting their text, have prolonged delays in starting a new sentence, and make more use of word suggestions throughout the day compared to healthy subjects (See Fig. 6a).

The EDSS aims to quantify the general disability of a pwMS, including motor and non-motor characteristics. For this target, the proposed model leverages four clusters of timing-related keystroke features. First, the model predicts a high level of disability for pwMS with high latency between keypresses and simultaneously holding the keys longer than usual. Furthermore, the model is prone to estimate a higher likelihood of EDSS values bigger than the sample median when the typing rhythms change over time without a regular pattern. Conversely, subjects who exhibit a fast and stable typing behaviour are more likely to have low EDSS score, hence a mild disability level See Fig. 6b).

Regarding the estimation of the upper limb function for pwMS, subjects who type slowly are more likely to require more time to complete the NHPT task. In the opposite direction, the faster the subject types, the less time it takes to complete the NHPT task, and by extension, the better the upper limb function. Further effects that drive the model prediction towards a higher probability of declining fine motor skills are an increment in typing speed change within days See Fig. 6c).

Finally, regarding the estimation of information processing speed, measured by SDMT, it was observed that pwMS who are fast in formulating sentences and capable of quickly correcting or adjusting their text are more likely to obtain a high score on the SDMT, which indicates adequate cognitive skills and vice-versa See Fig. 6d).

One can observe that the proposed wrapper-type feature selection strategy selects a different subset of features per target. However, the central value of the Fine Motor Score is chosen more frequently than the remaining composite scores, and it provides the highest impact on the model’s output for both NHPT and EDSS, but also relative to the clinical diagnosis between HC and pwMS. In Lam et al.^11^ it was shown that the keystroke features used to construct this cluster yielded very high test-rest reliability suggesting that these features can be considered an accurate representation of the participant’s performance and robust against irrelevant artefacts in the testing session such as environmental, psychological or methodological processes^34^.

Despite the model outputs’ being all significantly associated with their corresponding clinical outcome when demographic variables are taken into account, the effect of age had a higher impact in predicting the EDSS target than the algorithm-driven solely by keystroke dynamics (see Table 2). As mentioned in earlier sections, MS is a progressive disease; hence, the chance of having significant physical impairment increases as pwMS age, leading to a high EDSS score.

**Figure 7:**
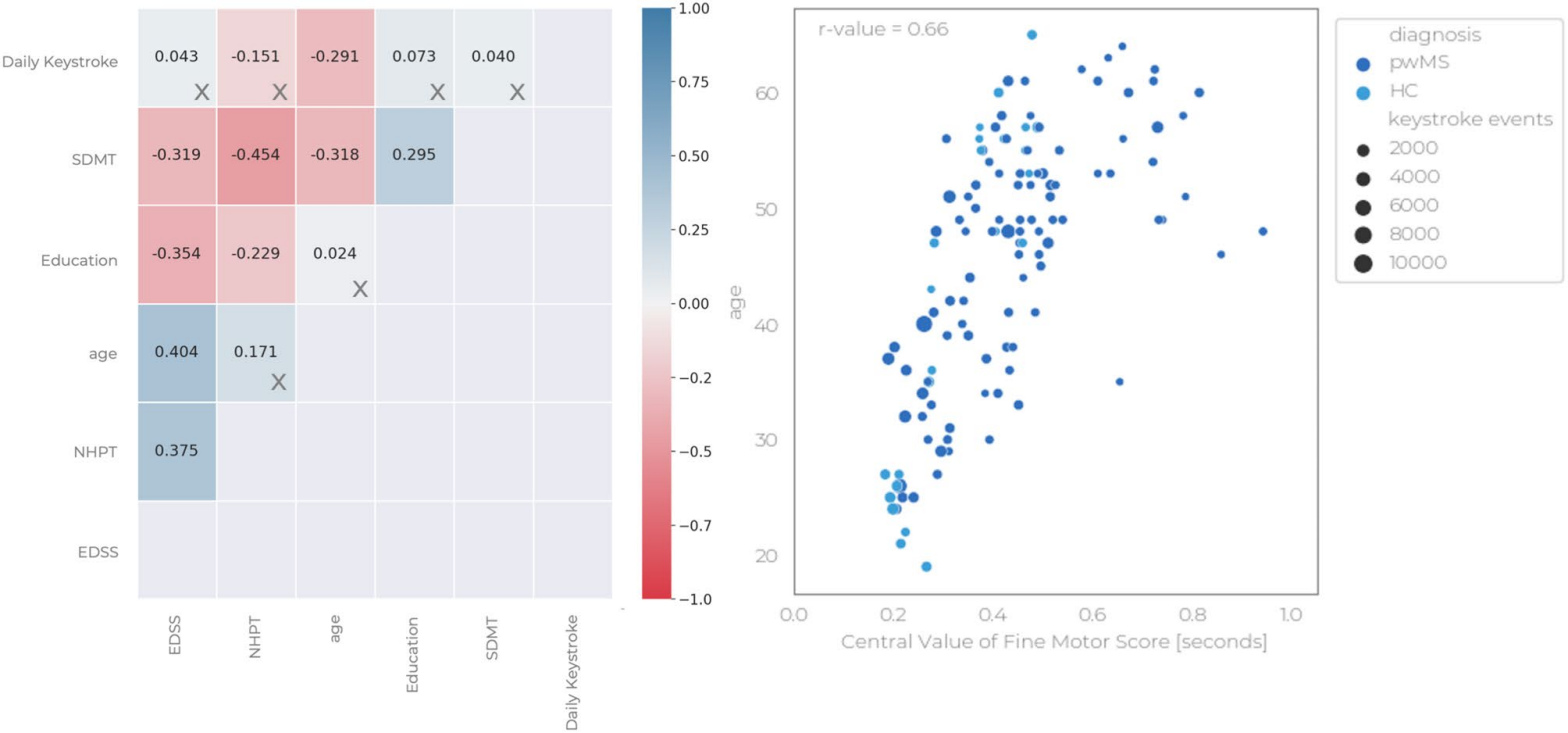
On the left is the correlogram relative to the clinical outcomes actual score measured at baseline, the corresponding daily keystroke events averaged within a two-week time window centred at *m*00, and the numerical demographics variables. A cross in the bottom-right quadrant denotes a non-significant* r* value with* p* value > 0.05. One can observe a non-negligible linear relationship between clinical outcomes and demographic variables. On the right is the scatter-plot between age and the most recurring composite score, i.e. the central value of the fine motor score, collected at baseline for both HC and pwMS within the same time range as previously mentioned. The dot size is tuned based on the average daily keystroke event. For the sake of visualization, the *x*-axis was set between 0 and 1 second, though an additional instance was recorded from an MS patient with pair $(x=1.68, y = 59)$ and average daily keystroke event count equal to 646.

An additional analysis was carried out to study the association between age and keystroke features. This analysis showed that timing-related features strongly correlate with age, suggesting that older pwMS tend to type slower. These findings are in line with the results of Salthouse^35^, which described the effect of age on keystroke dynamics and reported that older people had a slower tapping rate. Figure 7 shows the correlation values between the clinical scores, demographic variables and daily keystroke events, as well as the scatter-plot between age and the most recurring predictor, i.e., the central value of the fine motor score. Finally, the correlation values of the remaining composite scores w.r.t age are provided in Table A3.

With regards to the level of education, mild correlations have been observed with both EDSS and NHPT. Such an outcome is in line with other studies where similar relationships were found between literacy and various disease severity scales within an MS population^31,36–38^. More information regarding the education demographics can be found in the supplementary material Table A4.

**Figure 8:**
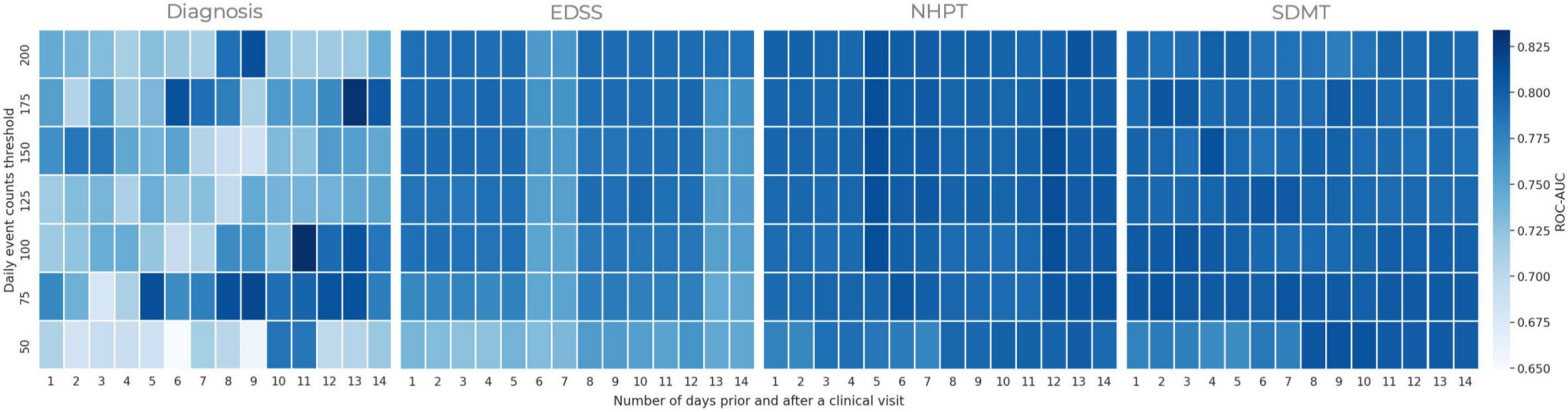
In this framework, the threshold value $\tau _{d}$ masks the daily aggregation of keystroke features with low typing events and the number of days prior to, and after a clinical visit, $w_{d}$ are treated as hyperparameters. For each clinical outcome, the best classification pipeline is tuned using an exhaustive grid search approach with AUC-ROC as the performance metric. The heatmap visualizes the hyper-parameter space where each square represents the cross-validated AUC-ROC score. One can observe a uniform pattern for both the NHPT and SDMT target. Conversely, the heat-map relative to the clinical diagnosis (HC versus pwMS) shows a less clear pattern, though the highest performance region is clustered around the bottom-right quadrant. Finally, as far as the EDSS is concerned, the hyper-parameter space suggests selecting a threshold of $\tau _{d} \ge 100$.

During the model design phase, one of the requirements was to determine the number of consecutive days to feed to the classification pipeline, as also shown in Fig. 4. In Lam et al.^13^ It was considered a 28-day (clinical visit $\pm 14$ days) and 14-day (clinical visit $\pm 7$ days) aggregation period for the fine motor and cognitive clusters, respectively, under the assumption that fine motor and cognitive functions are stable within such time windows. Furthermore, a keystroke event count threshold of 50 events was used to remove days with insufficient data. In this work, both the time window $w_{d}$ and the event count threshold $t_{d}$ were considered hyper-parameters and optimised for each clinical outcome with respect to the AUC-ROC metric. This data-driven approach resulted in the following optimal pairs $( \mathrm {w_{d}}, \mathrm {t_{d}} )$ equal to (11, 100), $(\pm 10, 125)$, $(\pm 12, 125)$ and $(\pm 4, 150)$ for the clinical diagnosis, EDSS, NHPT and SDMT, respectively. Figure 8 illustrates the mean AUC-ROC for all pairs $( \mathrm {w_{d}}, \mathrm {t_{d}} )$ considered in the grid search, and for this application, no substantial changes in terms of performance were observed for the EDSS, NHPT and SDMT prediction. Conversely, the prediction performance relative to the clinical diagnosis appeared more sensitive to such parameters with no clear pattern across the grid. Previously, studies regarding keystroke dynamics obtained through smartphone interactions have been primarily conducted in a laboratory environment, in which participants were asked to transcribe standardised text excerpts, or a specific type of smartphone was provided^7,39,40^. Contrary to laboratory studies, in this real-world study, data was collected during day-to-day use of smartphones. Collecting data in real-world settings allows for insight into the performance of patients in their daily life and enables researchers to go beyond data gathered during clinical visits. The findings of this study show the potential of keystroke data collected in the real world in providing insight into the performance of patients in between clinical visits and thus could be used to inform disease management strategies. However, future studies are needed to study the relationship between typing behaviour in relation to relapses and contrast-enhancing lesions while considering signal interferences from other sources, including behaviour (e.g. typing style), possible language related differences and technological aspects (e.g. smartphone-related aspects).

## Supplementary Information

Supplementary Information.

## Data Availability

The data that support the findings of this study are available from the corresponding author but restrictions apply to the availability of these data, which were used under license for the current study, and so are not publicly available. Data are however available from the authors upon reasonable request and with permission of the corresponding author.
