# Supplementary material for: Disease severity classification using passively collected smartphone-based keystroke dynamics within multiple sclerosis
Source: Sci Rep. 2023 Feb 1;13:1871. doi: 10.1038/s41598-023-28990-6 (PMC9892592; doi:10.1038/s41598-023-28990-6)
Supplement: Supplementary file 1 — Supplementary Information. [file 41598_2023_28990_MOESM1_ESM.pdf]

SUPPLEMENTARY MATERIAL

Aggregation	Description
Approximate Entropy	A measure of the unpredictability of fluctuations in a sequence[1].
Absolute sum of changes	The sum over the absolute value of consecutive changes in the series x computed as $\sum_i \mid x_{i+1} - x_i \mid$.
Maximum	The maximum value of a sequence.
Mean	The average value of a sequence.
Median	The middle number in a sorted sequence.
Minimum	The minimum value of a sequence.
Standard deviation	The average of the amount of variation or dispersion.

Table A1. Summary statistics used to aggregate keystroke sequences on a daily level.

Feature	Symbol	Description
Flight Time	FT	the time between a key being released and the next key press.
Hold Time	HT	the time for which a key is pressed.
Press Press Latency	PPL	the time between successive key presses.
Release Release Latency	RRL	the time between successive key releases.
Pre Correction Slowing	PreCS	the time between a backspace keystroke and the subsequent keystroke event.
Post Correction Slowing	PostCS	the time between a keystroke and a backspace event.
After Punctuation Pause	APP	the pause a user makes following the use of a punctuation key.

Table A2. Summary of keystroke features.

Composite Score	Feature	Aggregation	r(cluster, age)
Central value of Fine Motor Score	FT, PPL, RRL	Mean, Median	0.660^*_b
Absolute change of Fine Motor Score	FT, PPL, RRL	Absolute sum of changes	-0.057_b
Approximate entropy of Fine Motor Score	FT, PPL, RRL	Approximate Entropy	0.450^*_a
Standard deviation of Fine Motor Score	FT, PPL, RRL	Standard deviation	0.492^*_b
Minimum value of Fine Motor Score	FT, PPL, RRL	Minimum	0.470^*_a
Central value of Mutual Fine Motor Score	HT	Mean, Median	0.004_a
Central value of Cognition Score	PreCS, PostCS, APP	Mean,Median	0.569^*_a
Standard deviation of Cognition Score	PreCS, PostCS, APP	Standard deviation	0.405^*_a
Maximum value of Cognition Score	PreCS, PostCS, APP	Maximum	0.027^*_a
Minimum value of Cognition Score	PreCS, PostCS, APP	Minimum	0.510^*_b

Table A3. Cluster composition and corresponding correlation with age relative to pwMS, averaged within a two-week time window centred at baseline.
Glossary: FT: Flight Time; RRL: Release-Release Latency; PPL: Press-Press Latency; HT: Hold Time; PreCS: Pre-Correction Slowing; PostCS: Post-Correction Slowing; APP: After Punctuation Pause.
a Pearsons correlation coefficient.
b Spearmans log-rank correlation coefficient.
* $p < 0.05$.

Education level	Category	Amount	Description (in Dutch)
Low	1	0	Minder dan 6 klassen lagere school/basisschool niet afgemaakt.
Low	2	0	6 klassen lagere school/basisschool afgemaakt.
Low	3	1	Meer dan 6 klassen lagere school zonder afgesloten speciale opleiding/basisschool afgemaakt en verdere vervolgopleiding minder dan 2 jaar (VGLO, VBO, 1 of 2 jaar LTS/ULO/MAVO/Atheneum.
Low	4	4	Lager beroepsonderwijs (LTS A en B niveau/LEAO/LHNO/MAVO-3/3 achtereenvolgende jaren middelbaar algemeen voortgezet onderwijs en overgangsrapport naar jaar 4).
Low	5	37	Middelbaar algemeen voortgezet onderwijs en middelbaar beroepsonderwijs (MULO/MAVO-4/MBO/MTS/MEAO/MGDO/LTS C-niveau/LHNO C-niveau/3 achtereenvolgende jaren voorgezet algemeen onderwijs en overgangsrapport naar jaar 4).
Middle	6	57	Hoger beroepsonderwijs en voortgezet algemeen onderwijs (HAVO/Atheneum/ Gymnasium/HEAO/HBS/HBO/HTS/ Hogere landbouwschool/Hogere textielschool diploma).
High	7	27	Universitair onderwijs (Universiteit/TH/landbouwhogeschool diploma).

Table A4. Educational structure of the population[2].

References

1. Pincus, S. Approximate entropy (apen) as a complexity measure. *Chaos: An Interdiscip. J. Nonlinear Sci.* **5**, 110–117 (1995).

2. Lam, K.-H. *et al.* The use of smartphone keystroke dynamics to passively monitor upper limb and cognitive function in multiple sclerosis:a longitudinal analysis. *J Med Internet Res 2022;0(0):e0* DOI: 10.2196/37614 (in press).
